# Role of ARRB1 in prognosis and immunotherapy: A Pan-Cancer analysis

**DOI:** 10.3389/fmolb.2022.1001225

**Published:** 2022-09-23

**Authors:** Yingquan Ye, Haili Jiang, Yue Wu, Gaoxiang Wang, Yi Huang, Weijie Sun, Mei Zhang

**Affiliations:** ^1^ Oncology Department of Integrated Traditional Chinese and Western Medicine, The First Affiliated Hospital of Anhui Medical University, Hefei, China; ^2^ The Traditional and Western Medicine (TCM)-Integrated Cancer Center of Anhui Medical University, Hefei, China; ^3^ Department of Infectious Diseases, The First Affiliated Hospital of Anhui Medical University, Hefei, China

**Keywords:** ARRB1, pan-cancer, prognosis, tumor immunity, biomarker

## Abstract

**Background:** β-arrestin1 (ARRB1), was originally identified as a multifunctional adaptor protein. Although ARRB1 has recently been shown to also play an important role in tumor growth, metastasis, inflammation, and immunity, its relationship with distinct tumor types and the tumor immune microenvironment remains unclear.

**Methods:** We analyzed the ARRB1 expression profile and clinical characteristics in 33 cancer types using datasets from The Cancer Genome Atlas (TCGA) database. Clinical parameters such as patient survival, tumor stage, age, and gender were used to assess the prognostic value of ARRB1. The Human Protein Atlas (HPA) database was used to explore ARRB1 protein expression data. ESTIMATE and CIBERSORT algorithms were performed to assess immune infiltration. Furthermore, putative correlations between ARRB1 and tumor-infiltrating immune cells, the signatures of T-cell subtypes, immunomodulators, the tumor mutation burden (TMB), Programmed cell death ligand 1 (PD-L1), and microsatellite instability (MSI) were also explored. Gene functional enrichment was determined using GSEA. GSE40435 and GSE13213 cohorts were used to validate the correlation of ARRB1 with KIRC and LUAD clinicopathological parameters. Finally, the relationship between ARRB1 and immunotherapeutic responses was assessed using three independent immunotherapy cohorts, namely, GSE67501, GSE168204, and IMvigor210.

**Results:** We found that ARRB1 expression levels were lower in 17 tumor tissues than in the corresponding normal tissues. We further found that ARRB1 expression was significantly correlated with tumor stage in BRCA, ESCA, KIRC, TGCT, and THCA, while in some tumors, particularly KIRC and LUAD, ARRB1 expression was associated with better prognosis. ARRB1 expression was also positively correlated with the stromal score or the immune score in some tumors. Regarding immune cell infiltration, ARRB1 expression in DLBC was positively correlated with M1 macrophage content and negatively correlated with B-cell infiltration. Additionally, there was a broad correlation between ARRB1 expression and three classes of immunomodulators. Furthermore, high ARRB1 expression levels were significantly correlated with some tumor immune-related pathways. Finally, ARRB1 expression was significantly associated with MSI, PD-L1, and TMB in some tumors and with the efficacy of immune checkpoint inhibitors (ICIs) in melanoma.

**Conclusion:** ARRB1 has prognostic value in malignant tumors, especially in KIRC and LUAD. At the same time, ARRB1 was closely correlated with the tumor immune microenvironment and indicators of immunotherapy efficacy, indicating its great potential as a reliable marker for predicting the efficacy of immunotherapy.

## Introduction

Beta-arrestin1 (ARRB1), belonging to the arrestin family of proteins, was originally identified as an adaptor protein that regulates G protein-coupled receptor (GPCR) desensitization and internalization (Kang et al., 2005). ARRB1 is now known to be a ubiquitous adaptor protein that plays a role in the regulation of cell proliferation, differentiation, autophagy, and apoptosis (Yang et al., 2015; Das et al., 2016; Zhan et al., 2016; Lei et al., 2021). In addition to cytoplasmic functions, the nuclear activity of ARRB1 has recently been reported (Hoeppner et al., 2012; Purayil et al., 2015) and is thought to be a nuclear transcriptional regulator of the endothelin-1-induced β-catenin signaling (Rosanò et al., 2013). Meanwhile, ARRB1 can also serve as a scaffold for various signaling networks, including the Wnt, ERK1/2, and NF-κB signaling pathways (Cianfrocca et al., 2014; Rosano et al., 2014; Ko et al., 2021).

In addition, ARRB1 influences the development and biological function of T lymphocytes. There is evidence to support that ARRB1 plays an essential role in CD4^+^ T-cell survival through promoting an increase in histone H4 acetylation at the Bcl-2 promoter region and thereby enhancing its transcription (Shi et al., 2007). ARRB1 is also involved in the regulation of T-cell activation. The binding of the T-cell receptor (TCR) to its cognate major histocompatibility complex (MHC) stimulates the production of cyclic adenosine monophosphate (cAMP), which activates the inhibitory PKA-Csk1 pathway and negatively feeds back to inhibit T-cell activation (Sharma and Parameswaran, 2015). These observations suggest that ARRB1 promotes T-lymphocyte development and enhances T-cell-mediated immune responses, suggesting that it may be involved in regulating the tumor immune microenvironment. Moreover, ARRB1 plays an important role in tumor cell epithelial-to-mesenchymal transition (EMT) (Kong et al., 2018; Song et al., 2020; Song et al., 2021) and metabolic reprogramming (Mamouni et al., 2021), and also regulates important signaling pathways in tumors (Bagnato and Rosanò, 2019). Overall, ARRB1 has an important role in both the tumor and immune microenvironment. However, few studies have focused on investigating the immunotherapeutic value of ARRB1 in human pan-cancer.

In the present study, we explored the ARRB1 expression profile in 33 cancer types and investigated the potential impact of ARRB1 on prognosis for survival in cancer and the tumor immune microenvironment. The correlation of ARRB1 with stromal cells, immune cells, and immune modulators (immunosuppressants, immunostimulants, and MHC molecules) in the tumor immune microenvironment were all investigated. In addition, correlations between ARRB1 and predictors of immune checkpoint inhibitor (ICI) efficacy (tumor mutation burden [TMB], microsatellite instability [MSI], and Programmed cell death-ligand 1 [PD-L1]). We further validated the relationship between ARRB1 and responses to immunotherapy in different clinical cohorts. In conclusion, our results contribute to elucidating the immunotherapeutic role of ARRB1 in cancer and may serve as a reference for future functional experiments both *in vivo* and *in vitro*.

## Materials and methods

### Data collection

The University of California at Santa Cruz Xena website (https://xenabrowser.net/) was utilized to collect ARRB1 data for 33 cancer types from The Cancer Genome Atlas (TCGA) database (http://cancergenome.nih.gov), including genomic and clinicopathological data. The abbreviations and full names of the cancers involved in the study are presented in Table 1. Data for somatic mutations were gathered from TCGA database. Strawberry Perl (version 5.32.1.1) was used to merge ARRB1 expression data obtained from TCGA database, after which an expression data matrix was constructed for further analysis. In addition, the Human Protein Atlas (HPA) database (https://www.proteinatlas.org) was employed to explore ARRB1 protein expression data for various cancer types, and obtain images of ARRB1 protein immunohistochemical staining in cancer tissues and corresponding normal tissues. Expression and clinical data for renal cell carcinoma (ID: GSE67501, GSE40435), lung adenocarcinoma (ID: GSE13213) and metastatic melanoma (ID: GSE168204) cohorts were downloaded from the Gene Expression Omnibus (GEO) database (https://www.ncbi.nlm.nih.gov/geo/). GSE67501 and GSE168204 were performed for immunotherapy efficacy analysis, and the other two were applied for clinicopathological parameter correlation analysis. Data for the Advanced Urothelial Carcinoma cohort (IMvigor210) was obtained from previously published studies (Mariathasan et al., 2018).

**TABLE 1:** 33 types of human cancers employed in our research.

Abbreviation	Full name
ACC	Adrenocortical carcinoma
BLCA	Bladder urothelial carcinoma
BRCA	BRCA Breast invasive carcinoma
CESC	Cervical squamous cell carcinoma and endocervical adenocarcinoma
CHOL	Cholangiocarcinoma
COAD	Colon adenocarcinoma
DLBC	Lymphoid neoplasm diffuse large B-cell lymphoma
ESCA	Esophageal carcinoma
GBM	Glioblastoma multiforme
HNSC	Head and neck squamous cell carcinoma
KICH	Kidney chromophobe
KIRC	Kidney renal clear cell carcinoma
KIRP	Kidney renal papillary cell carcinoma
LAML	Acute myeloid leukemia
LGG	Brain lower grade glioma
LIHC	Liver hepatocellular carcinoma
LUAD	Lung adenocarcinoma
LUSC	Lung squamous cell carcinoma
MESO	Mesothelioma
OV	Ovarian serous cystadenocarcinoma
PAAD	Pancreatic adenocarcinoma
PCPG	Pheochromocytoma and paraganglioma
PRAD	Prostate adenocarcinoma
READ	Rectum adenocarcinoma
SARC	Sarcoma
SKC	Skin cutaneous melanoma
STAD	Stomach adenocarcinoma
TGCT	Testicular germ cell tumors
THCA	Thyroid carcinoma
THYM	Thymoma
UCEC	Uterine corpus endometrial carcinoma
UCS	Uterine carcinosarcoma
UVM	Uveal melanoma

### Correlation between ARRB1 expression and clinical parameters or prognosis for survival

The differential expression of ARRB1 between tumor and normal tissues was analyzed using the “limma” package in R software (version 4.1.2), where the “ggpubr” package was used to plot the box diagrams. The correlation between ARRB1 mRNA expression and other clinical parameters (tumor stage, age, and gender) were also analyzed. In addition, to investigate the prognostic value of ARRB1 in pan-cancer, univariate Cox regression analysis was performed using the R packages “survminer” and “survival”. Overall survival (OS), progression-free survival (PFS), disease-specific survival (DSS), and disease-free survival (DFS) were all analyzed. *p* value and hazard ratios (HR) with 95% confidence intervals (CI) were ascertained for each cancer type. Forest plots were generated using the R package “forestplot”.

### Analysis of the correlation between ARRB1 expression and components of the tumor microenvironment

ESTIMATE is a technique used to estimate the number of infiltrating immune cells and stromal cells in tumor tissue (Yoshihara et al., 2013). Here, the number of immune cells and stromal cells in the tumor tissue of each case were calculated using the “ESTIMATE” and “limma” packages in R to obtain the immune score and stromal score. The sum of the immune score and stromal score is the ESTIMATE Score, which is inversely correlated with tumor purity. Subsequently, putative correlations between ARRB1 expression and immune and stromal scores in different tumors were evaluated. In addition, the CIBERSORT algorithm in R was employed to estimate the abundance of immune cell infiltration in each case, and its correlation with ARRB1 expression in different tumors was further analyzed referring to the method used to assess tumor-infiltrating lymphocyte subpopulations in Newman et al. (Newman et al., 2015). The R “ggplot2”, “ggpubr”, and “ggExtra” packages were used to plot correlation graphs for results that met the thresholds (**|**Pearson correlation coefficient**|** > 0.3 and *p* < 0.01).

The Tumor-Immune System Interactions Database (TISIDB) (http://cis.hku.hk/TISIDB/index.php) is a portal for the analysis of tumor/immune system interactions that integrates multiple heterogeneous data types for the analysis of targeted gene and immune signature relationships in various tumors. Potential relationships between ARRB1 expression and three types of immunomodulators (immunosuppressants, immunostimulants, and MHC molecules) were explored through the TISIDB web portal. The six results with the highest absolute values of correlation coefficients for each of these immunomodulators are displayed in plots.

### Gene set enrichment analysis

GSEA is a computational method based on existing knowledge of the biological significance, function, and location of genes and is used to build a database containing multiple functional genomes (Subramanian et al., 2005). The biological functions of ARRB1 in different tumors were subjected to GSEA. The Gene Ontology (GO) gene set “c5.go.v7.4.symbols” gene set and the Kyoto Encyclopedia of Genes and Genomes (KEGG) gene set “c2.cp.kegg.v7.4.symbols” were downloaded from the GSEA website (https://www.gsea-msigdb.org/gsea/downloads.jsp). The samples were then divided into high and low expression groups based on the median ARRB1 expression; *p* < 0.05 was set as the significance threshold and the R packages “limma”, “enrichplot”, “clusterProfiler”, and “org.Hs.eg.db” were used to perform enrichment analysis and visualize the results.

### Analysis of the immunotherapeutic response

The TMB, defined as the total number of somatic mutations per megabase of genome examined (McNamara et al., 2020), has been used as a biopredictive marker for responses to ICI treatment in a variety of solid tumors (Samstein et al., 2019; Marabelle et al., 2020; Jiang et al., 2022) and correlates with tumor prognosis (Kang et al., 2020). Meanwhile, MSI leads to the accumulation of mutations that are potentially significant predictive biomarkers of ICI responses (Dudley et al., 2016). Furthermore, Immune checkpoint PD-L1 on cancer cells binds to programmed cell death-1 (PD-1) on immune cells and contributes to the tumor immune escape (Yi et al., 2021). In some cases, immunotherapy response correlates with tumor PD-L1 expression (Yi et al., 2018). To investigate the correlation between ARRB1 expression and the TMB, MSI or PD-L1, the R package “fmsb” was used to create a correlation radar map; the correlation between ARRB1 expression and immune efficacy was further validated *via* three independent immunotherapy cohorts. Referring to the methodology of Mo et al. (Mo et al., 2021), partial response and complete response cases in the cohort were categorized as the responder group and cases of stable disease and progressive disease as the non-responder group. Finally, the Wilcoxon test was used to determine the difference in ARRB1 expression between the two groups.

### Statistical analysis

R software (version 4.1.2) and attached packages was used for statistical analyses. The comparisons of between-group ARRB1 expressions were conducted by Wilcoxon test. The correlation concerning ARRB1 expression was analyzed by Spearman’s correlation test. Survival curves were plotted using the Kaplan-Meier method. The relationship between ARRB1 expression and survival was analyzed using the Cox proportional regression method to derive hazard ratios. Two-tailed *p* values <0.05 were considered significant.

## Results

### Analysis of ARRB1 expression in pan-cancer

**FIGURE 1:**
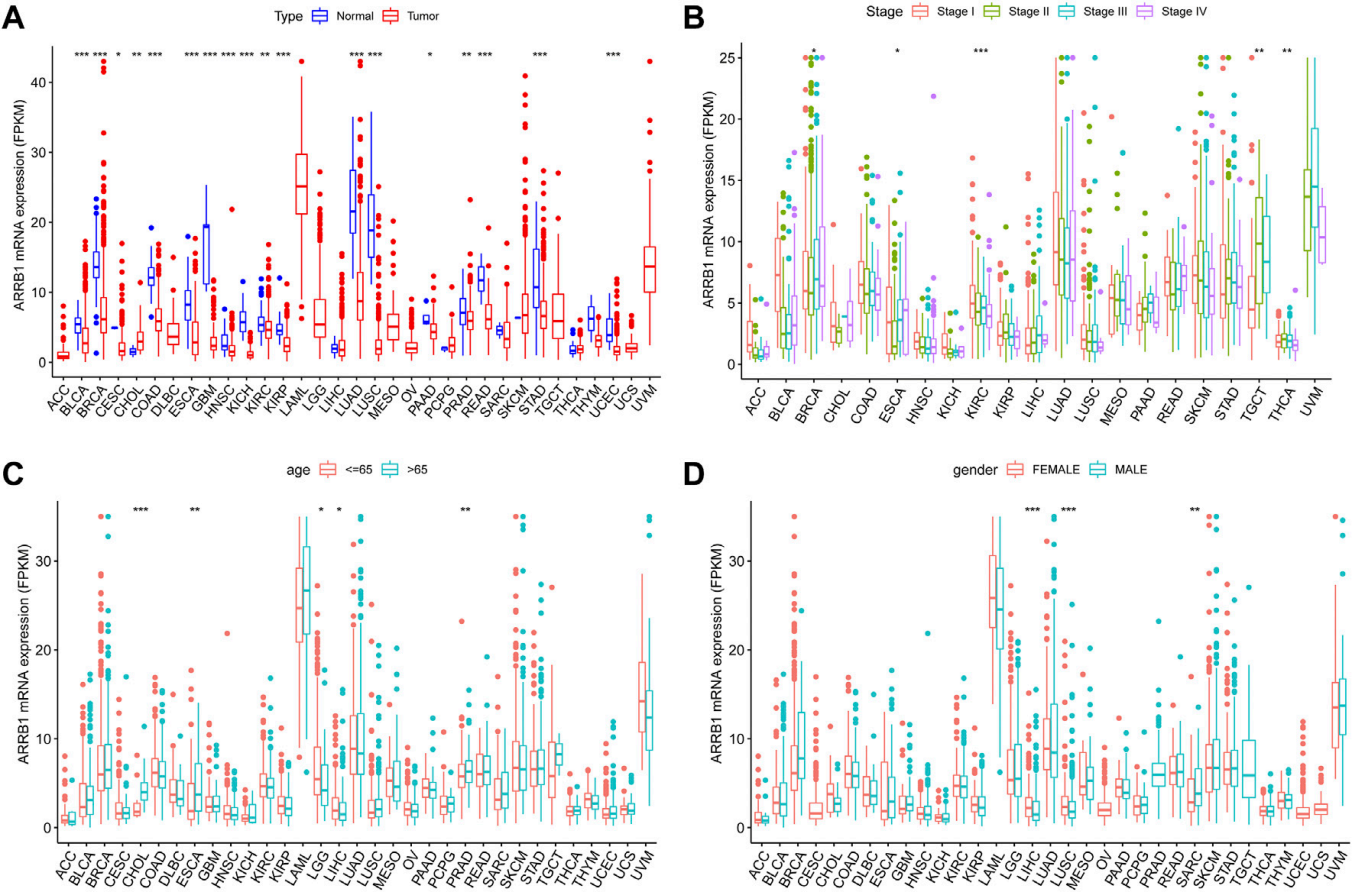
The clinical correlation of ARRB1. **(A)** The differential expression of ARRB1 mRNA between the tumor and normal groups in 33 cancer types. **(B)** ARRB1 mRNA expression levels in tumors of different stages. **(C)** ARRB1 mRNA expression levels in different age groups. **(D)** The correlation of ARRB1 mRNA expression levels with gender. *p < 0.05, **p < 0.01, and ***p < 0.001.

The expression level of ARRB1 mRNA in tumor tissues was lower than that in the corresponding control tissues in 17 of the 33 cancers assessed, including BLCA, BRCA, CESC, COAD, ESCA, GBM, HNSC, KICH, KIRC, KIRP, LUAD, LUSC, PAAD, PRAD, READ, STAD, and UCEC (Figure 1A). The ARRB1 expression level in tumor tissues was significantly higher than that in CHOL control tissues. ARRB1 expression was significantly correlated with tumor stage in BRCA, ESCA, KIRC, TGCT, and THCA (Figure 1B). Meanwhile, ARRB1 was highly expressed in elderly patients with CHOL, ESCA, and PRAD, which contrasted with the low expression seen in elderly patients with LGG and LIHC (Figure 1C). Additionally, the results revealed that ARRB1 expression was significantly higher in females than in males in LIHC and LUSC, whereas the opposite was observed in SARC (Figure 1D).

**FIGURE 2:**
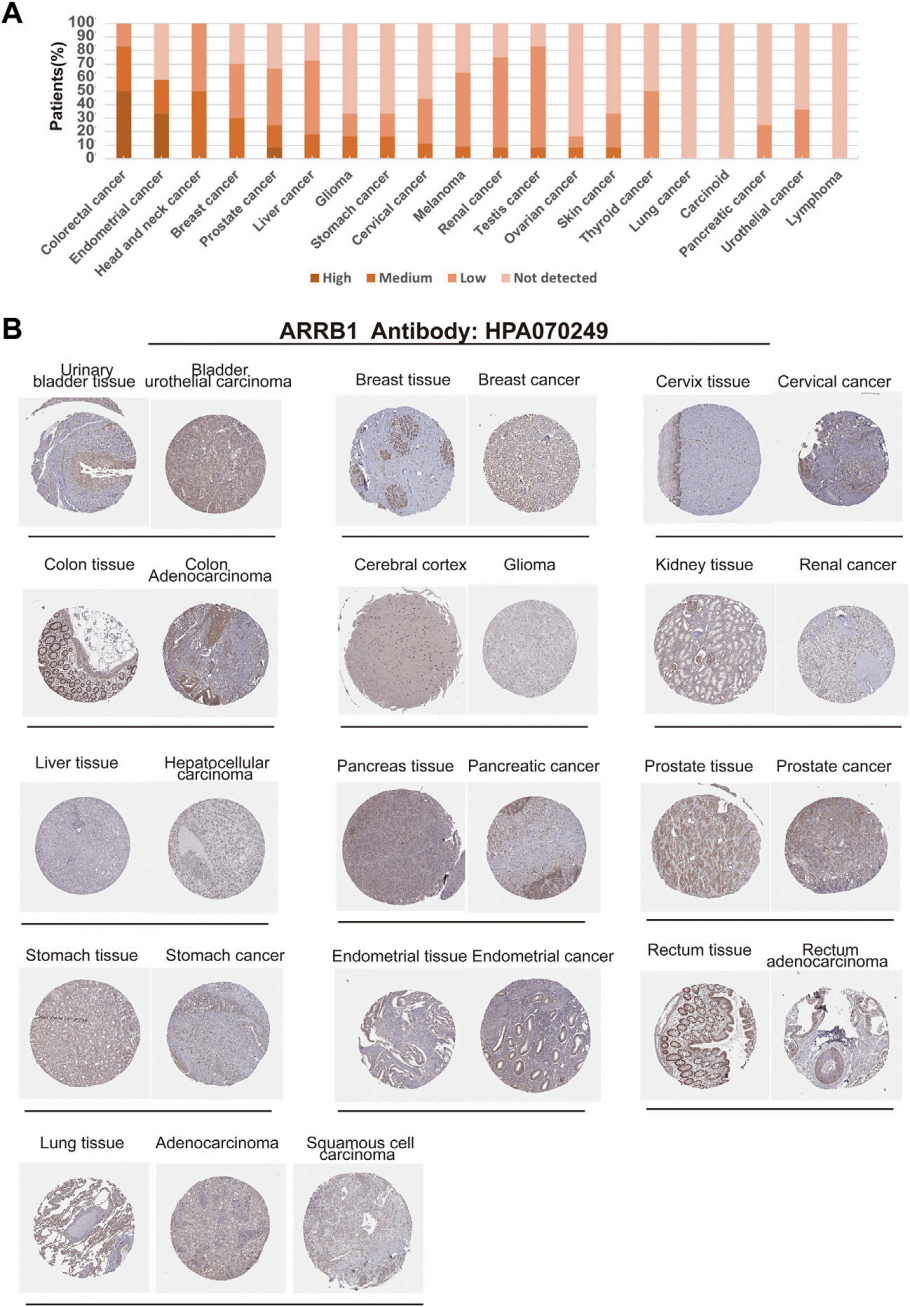
Expression of ARRB1 protein in various cancer tissues in the HPA database. **(A)** Percentage of patients with high, medium or low ARRB1 protein expression levels in different cancers in their respective tumors. **(B)** Immunohistochemical images of ARRB1 expression in various cancer tissues and corresponding normal tissues.

We further used HPA data to analyze the expression of ARRB1 protein in different cancer tissues and corresponding normal tissues. The results showed that ARRB1 protein expression was observed in 17 cancer types (Figure 2A). Among them, ARRB1 showed the highest percentage of expression in colorectal cancer, while showing lower levels of expression in most tumor tissues. Immunohistochemical staining results showed different expression levels of ARRB1 protein in 13 normal or tumor tissues (Figure 2B).

### The prognostic value of ARRB1 in pan-cancer

**FIGURE 3:**
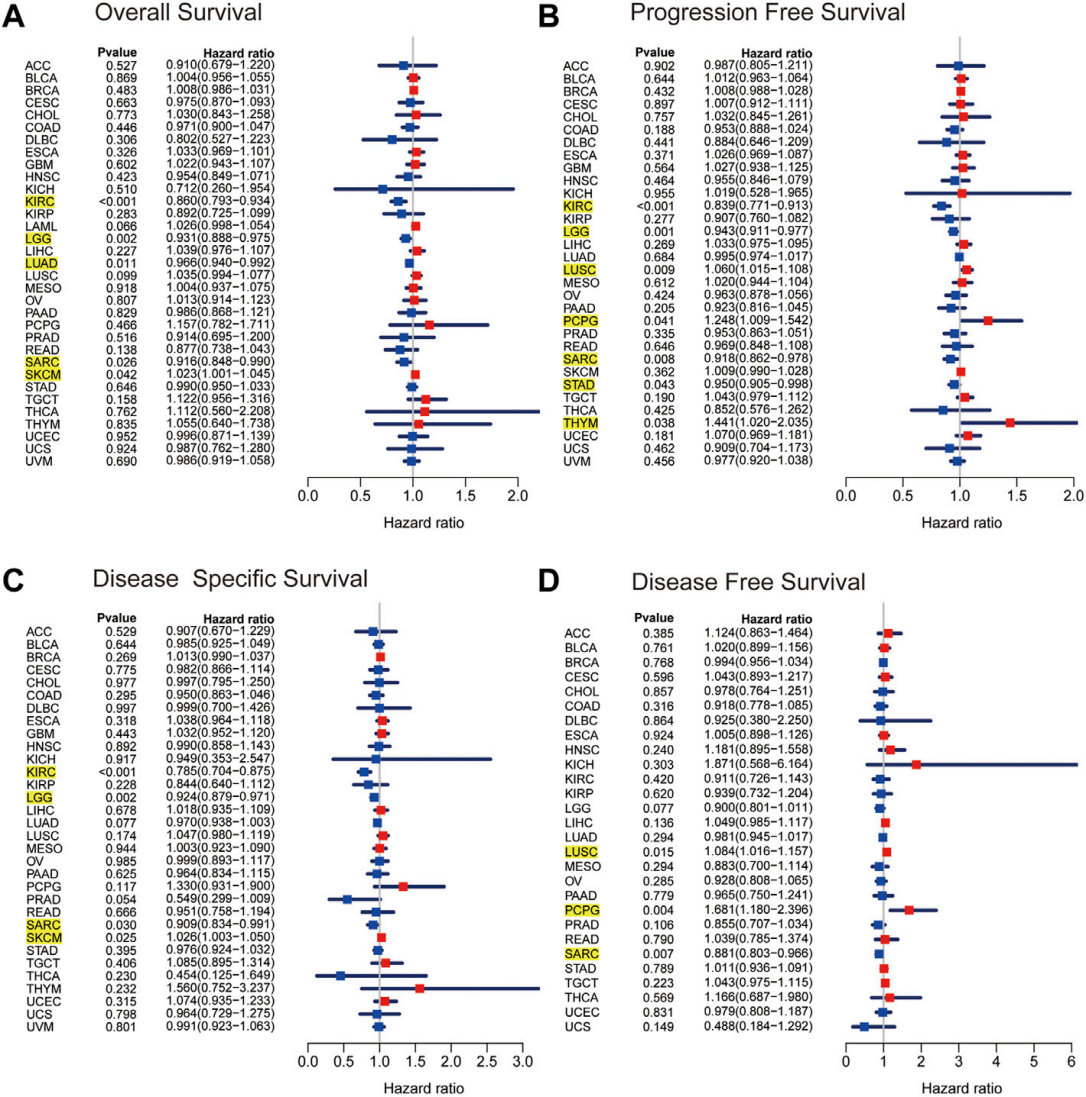
The Kaplan–Meier plots of univariate Cox regression analyses. Forest plots showing the associations between ARRB1 expression and **(A)** OS, **(B)** PFS, **(C)** DSS, and **(D)** DFS of patients in 33 cancers. The yellow highlighted section suggested that ARRB1 expression was significantly associated with prognosis in these cancers (*p* < 0.05).

**FIGURE 4:**
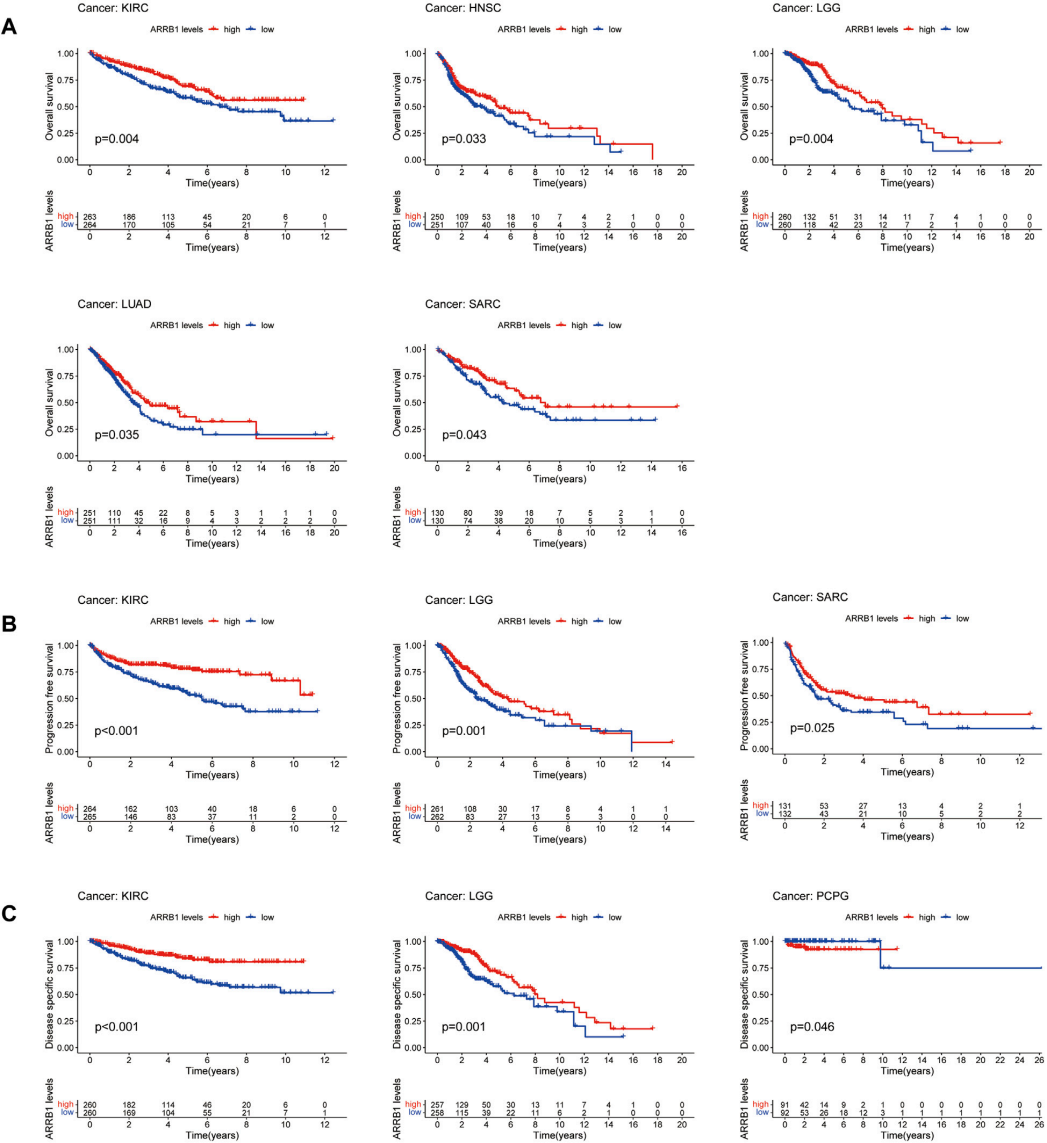
Associations of ARRB1 expression with survival among cancer patients. Kaplan-Meier survival curves showing **(A)** OS, **(B)** PFS, and **(C)** DSS in pan-cancer. Only significant outcomes were displayed (*p* < 0.05).

As a first step, we used a Cox proportional hazards regression model to analyze the association of ARRB1 expression with OS, PFS, DSS, and DFS in patients in pan-cancer. Analysis of the OS forest plot (Figure 3A) indicated that ARRB1 expression was protective against KIRC, LGG, LUAD, and SARC, whereas it was a risk factor in SKCM. Regarding PFS (Figure 3B), elevated ARRB1 expression showed a significant and positive association with PFS in KIRC, LGG, STAD, and SARC and a significant and negative association with outcome in LUSC, PCPG, and THYM. For DSS (Figure 3C), ARRB1 expression was protective against KIRC, LGG, and SARC, but appeared to be a risk factor in SKCM. Analysis of the DFS forest plot (Figure 3D) further confirmed the protective role of ARRB expression in SARC; however, ARRB expression was deemed to be a risk factor in LUSC and PCPG. Notably, all the above forest plots suggested that ARRB1 expression exerted a protective effect against SARC and was also associated with longer OS, PFS, and DSS in KIRC and LGG. In addition, Kaplan–Meier curve analysis showed that high ARRB1 expression predicted a good prognosis in KIRC, HNSC, LGG, LUAD, and SARC (Figure 4A). Meanwhile, high ARRB1 expression was associated with prolonged PFS in KIRC, LGG, and SARC (Figure 4B). Regarding DSS, high ARRB1 expression was equally favorable in KIRC and LGG (Figure 4C). Interestingly, the expression levels of ARRB1 were not significantly differential on the Kaplan–Meier DFS curve for DFS.

Furthermore, considering that ARRB1 is not only associated with a good prognosis in KIRC and LUAD patients, but is also lowly expressed in both tumor tissues. We validated the correlation between ARRB1 expression and clinicopathological parameters in GSE40435 and GSE13213 independent cohorts. The results showed that ARRB1 expression was relatively high in low-grade KIRC and low-stage LUAD (Supplementary Figure S1), further suggesting a potential relationship between ARRB1 and tumor prognosis and aggressiveness.

### Analysis of the association between ARRB1 expression and immune infiltration in pan-cancer

**FIGURE 5:**
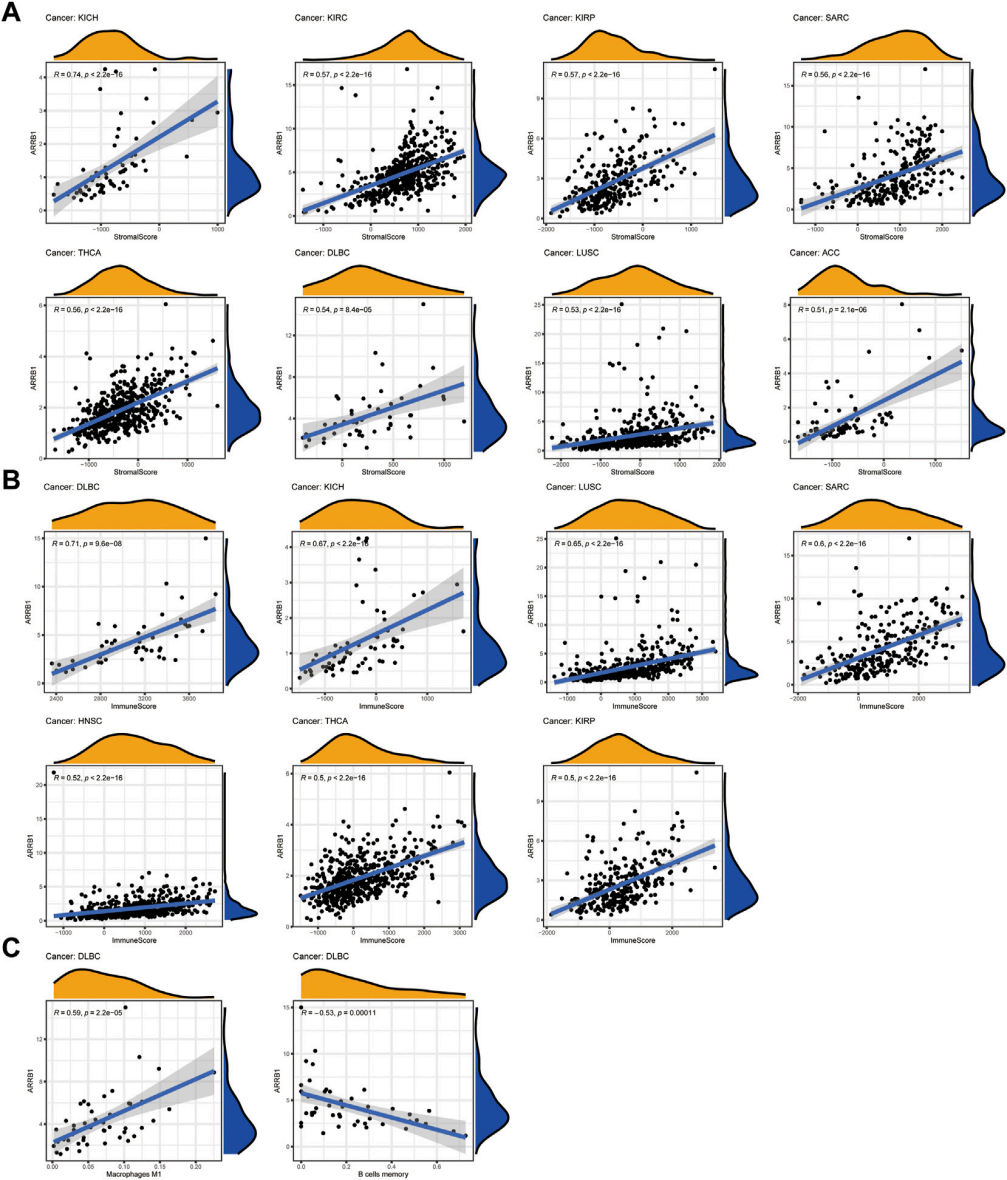
Correlation between ARRB1 expression and ESTIMATE score and immune cell infiltration. **(A)** Correlation between ARRB1 expression and stromal cell score in tumor tissue; **(B)** Correlation between ARRB1 expression and immune cell score in tumor tissue; **(C)** Correlation between ARRB1 expression and immune cell infiltration. Only correlation plots with correlation coefficients *R* > 0.5 and *p* < 0.001 are illustrated.

**FIGURE 6:**
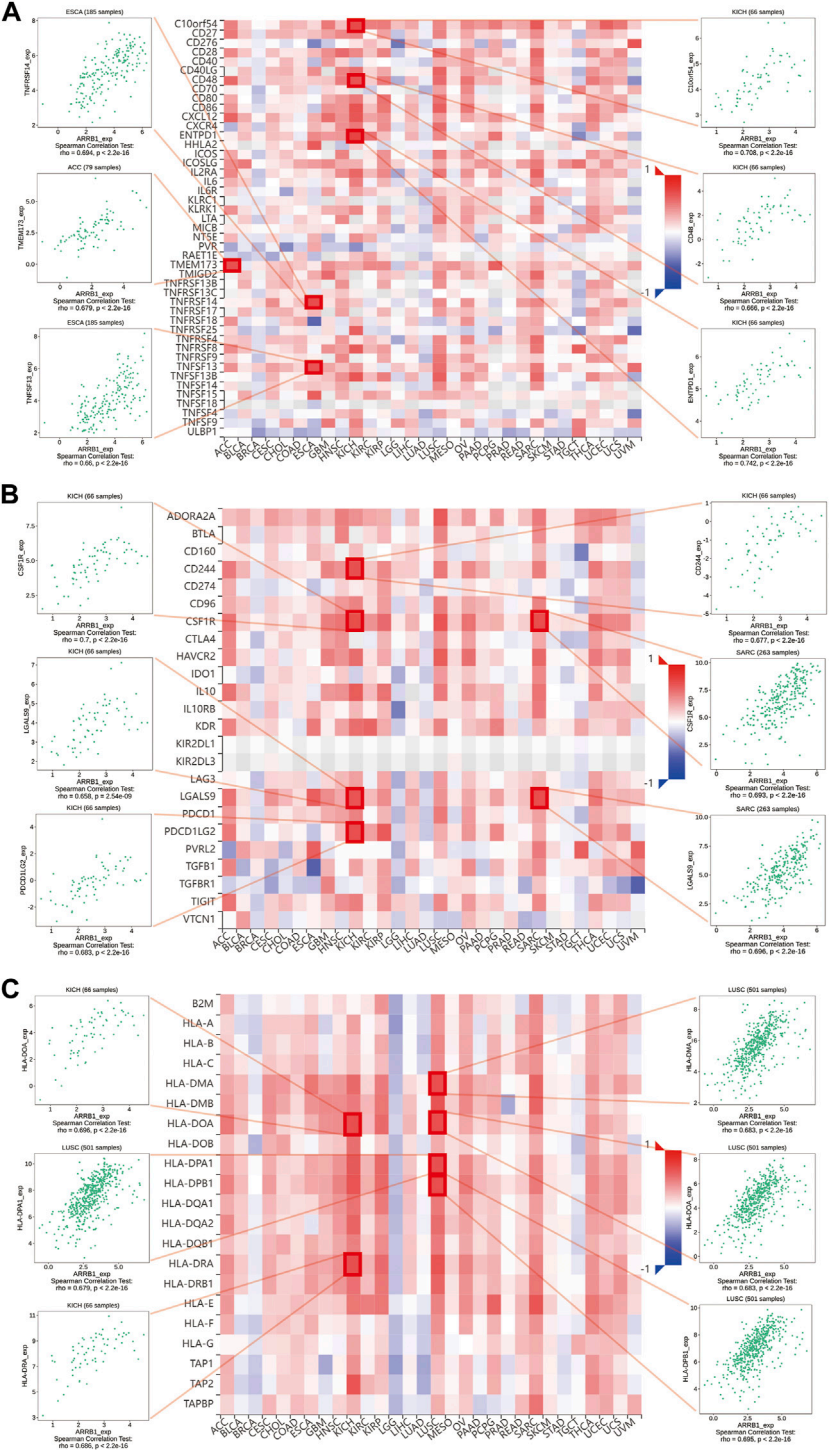
Association of ARRB1 with three kinds of immunomodulators across human cancers. **(A)** Association of ARRB1 expression with immunostimulators. **(B)** Association of immunoinhibitors with ARRB1 expression. **(C)** Correlation between ARRB1 expression and MHC molecules. Red color denotes positive correlation, while blue color represents negative correlation. Dot plots are used to show the top 6 strongest associations for each section.

Here, we first investigated the association of ARRB1 expression with the ESTIMATE score (the sum of the immune score and stromal score) and immune cell infiltration. The results show that ARRB1 expression was positively correlated with the stromal score in KICH, KIRC, KIRP, SARC, THCA, DLBC, LUSC, and ACC and with the immune score in DLBC, KICH, LUSC, SARC, HNSC, THCA, and KIRP (**|**R**|** ≥ 5) (Figures 5A,B). For immune cell infiltration, ARRB1 expression in DLBC was positively correlated with M1 macrophage content and negatively correlated with B-cell infiltration (**|**R**|** ≥ 5) (Figure 5C). In addition, the results of the correlation analysis with |R| < 5 are shown in Supplementary Figures S2, S3. Next, we investigated whether a correlation existed between ARRB1 expression and three categories of immunomodulators. The results showed that, among the 45 immunostimulants included in the analysis, ARRB1 expression was positively correlated with C10orf54, CD48, and ENTPD1 in KICH, as well as with TNFRSF14 and TNFSF13 in ESCA and TMEM173 in ACC (Figure 6A). Regarding immunosuppression (Figure 6B), ARRB1 expression was positively correlated with CD244, CSF1R, LGALS9, and PDCD in KICH and with CSF1R and LGALS9 in SARC. For MHC molecules (Figure 6C), dot plot analysis indicated that ARRB1 expression was positively correlated with HLA-DMA, HLA-DOA, HLA-DMA1, and HLA-DMA2 in LUSC and with HLA-DOA and HLA-DRA in KICH. Notably, ARRB1 expression was positively correlated with most MHC molecules in ACC, KICH, LUSC, OV, SARC, THCA, UCEC, and UCS, but was negatively correlated with most MHC molecules in LGG.

### GSEA relating to ARRB1 expression in pan-cancer

**FIGURE 7:**
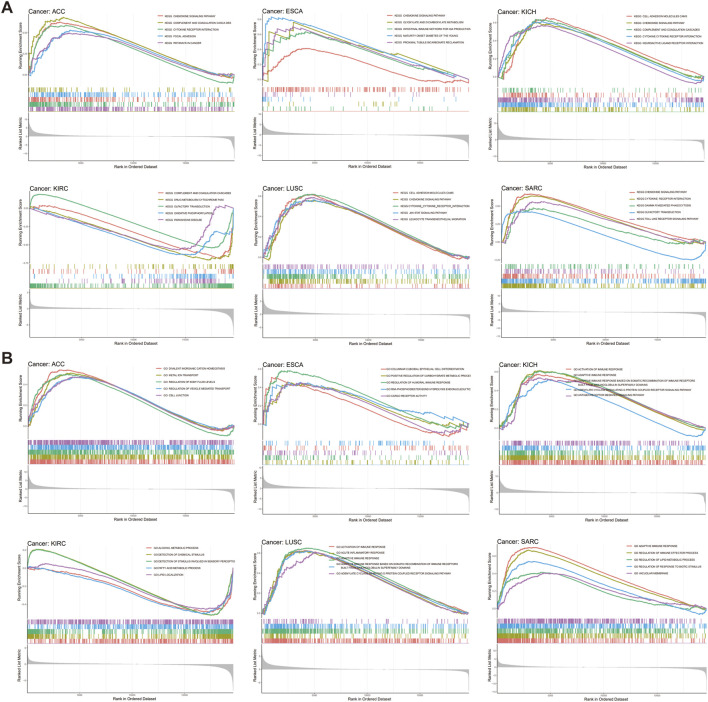
GSEA of ARRB1 expression in cancers. **(A)** GO enrichment plots by the ARRB1 expression from GSEA analysis. **(B)** KEGG enrichment plots by the ARRB1 expression.

Considering the significant connection identified between ARRB1 expression and tumor prognosis and immunity, GSEA was used to explore the potential biological implications and pathways associated with ARRB1 expression. As illustrated in Figure 7A, in terms of GO, high ARRB1 expression levels in LUSC and KICH were positively associated with activation of the immune response, adaptive immune response, adaptive immune response based on the somatic recombination of immune receptors built from immunoglobulin superfamily domains, and adenylate cyclase modulating G protein-coupled receptor signaling pathway. Adaptive immune response and regulation of immune effector processes also showed significant enrichment in SARC. In terms of KEGG (Figure 7B), ARRB1 was positively correlated with chemokine signaling pathways in ACC, ESCA, KICH, LUSC, and SARC, and cytokine receptor interaction in ACC, KICH, and LUSC. Furthermore, we observed that, in ESCA, the intestinal immune network pathway used for IGA production was enriched in the high ARRB1 expression group. In addition, in SARC, the Toll-like receptor signaling pathway, which is closely related to immunity, was enriched in the high ARRB1 expression group. Moreover, ARRB1 was positively enriched in immune-related biological processes in PCPG, THCA, CESC, LIHC, PAAD, GBM, BLCA, UCEC, CHOL and HNSC (Supplementary Figure S4). Meanwhile, we observed that the JAK-STAT pathway exhibited positive enrichment in THCA, TGCT, UCEC and PRAD (Supplementary Figure S5). Our findings suggested that ARRB1 generally correlated with many biological behaviors of cancer and plays different roles in different tumor types.

### Analysis of ARRB1 expression and responses to immunotherapy

**FIGURE 8:**
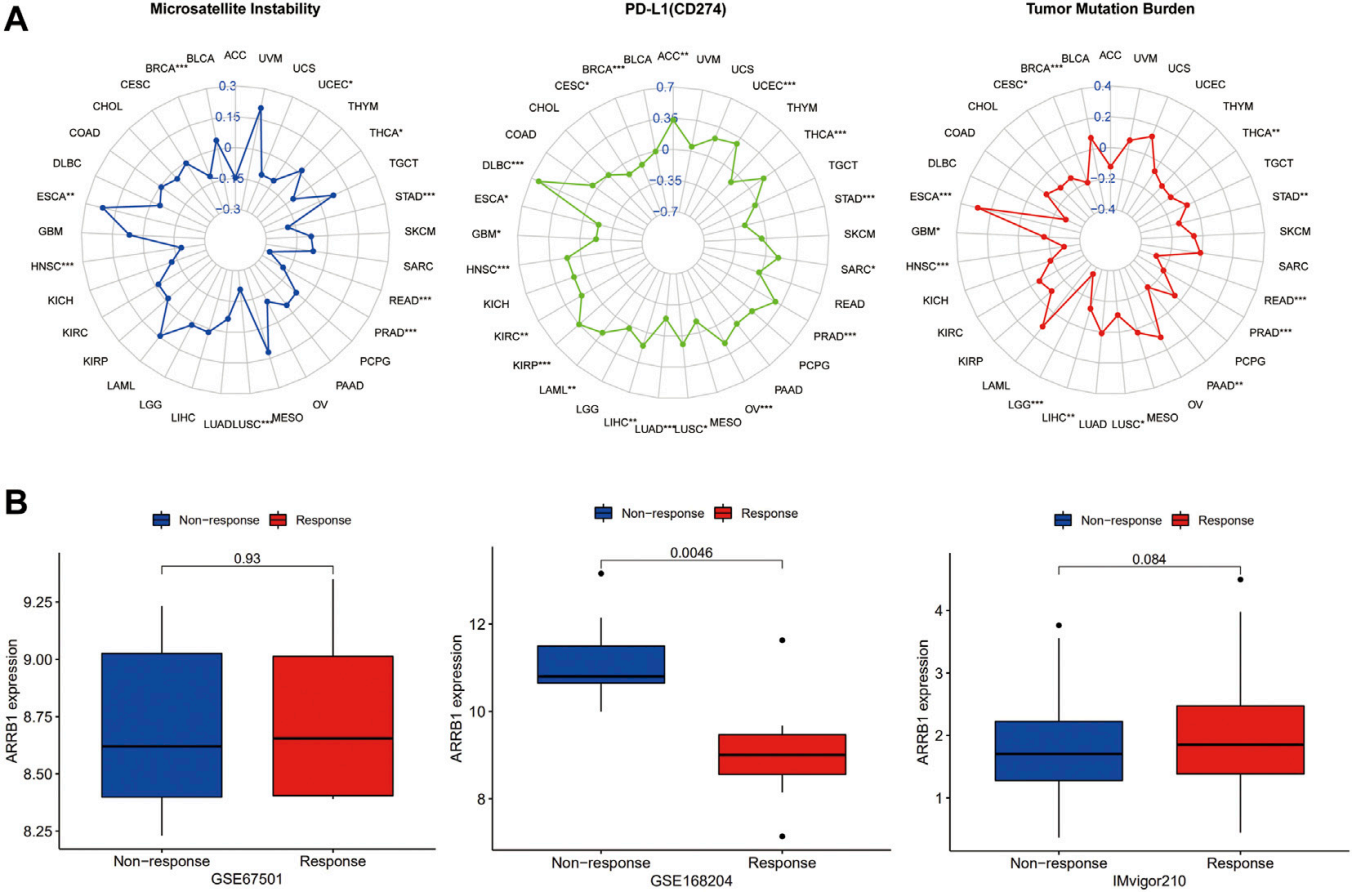
The correlation between ARRB1 expression and both predictive markers of immunotherapeutic efficacy and response to immunotherapy. **(A)** Radar chart of the relationship between ARRB1 expression and MSI, PDL1 (CD274), and TMB. **(B)** Relationship between ARRB1 expression and immunotherapeutic response. *p < 0.05, **p < 0.01, and ***p < 0.001.

Immunotherapy represents a breakthrough in the field of anticancer treatment. To investigate the correlation between ARRB1 expression and immunotherapeutic efficacy, we first sought to determine whether there was a correlation between the expression of ARRB1 and biomarkers for the prediction of immune checkpoint inhibitor efficacy (MSI, TMB and PD-L1). As shown in Figure 8A, ARRB1 expression was positively correlated with both MSI and TMB in ESCA, but was negatively correlated with these biomarkers in THCA, STAD, READ, PRAD, LUSC, HNSC, and BRCA. Moreover, ARRB1 expression was also negatively correlated with MSI in UCEC. A negative correlation between the TMB and ARRB1 expression was also found in PAAD, LIHC, LGG, GBM, and CESC. In terms of PD-L1, its expression in ACC, UCEC, THCA, SARC, PRAD, OV, LUSC, LIHC, LAML, KIRP, KIRC, HNSC and DLBC was positively correlated with the ARRB1 mRNA expression, while it was the opposite in STAD, LUAD, GBM, ESCA, CESC and BRCA. Finally, we investigated the relationship between ARRB1 expression and immunotherapeutic responses in three independent tumor immunotherapy cohorts. We found that, in all three cohorts, ARRB1 expression was significantly higher in the non-responder group than in the responder group of the PD1 blockade cohort in metastatic melanoma; however, no significant difference in ARRB1 expression was detected between the responder and non-responder groups of the nivolumab treatment of renal cell carcinoma and atezolizumab intervention in advanced urothelial carcinoma cohorts (Figure 8B).

## Discussion

ARRB1 was originally identified as a regulator of GPCR signaling and there is growing evidence that it is widely involved in the biological processes of cell proliferation, invasion, migration, angiogenesis, and drug resistance in tumors (Rosano and Bagnato, 2016). In this study, we undertook a comprehensive analysis of differential ARRB1 expression among 33 tumor types and normal tissues, and identified the clinical prognostic value and potential immunotherapeutic value of ARRB1 in a variety of tumors. By analyzing the potential relationship between ARRB1 expression and the prognosis of tumor patients in human pan-cancer, we first observed that in differentially expressed tumors, except for CHOL, ARRB1 expression was significantly higher in 17 normal tissues than in tumor tissues. Although ARRB1 expression was not associated with tumor-related clinical parameters such as a TNM stage, age, or gender in most tumors, we still observed some meaningful phenomena. Notably, we found that the TNM stage was significantly correlated with ARRB1 expression in KIRC, suggesting that ARRB1 may be associated with the malignancy and aggressiveness of KIRC. In addition, ARRB1 expression was significantly higher in normal tissue than in breast cancer tissue, as well as in higher-stage tumors relative to lower-stage ones. Son et al. (Son et al., 2019) reported that ARRB1 overexpression reduced the growth and migratory potential of triple-negative breast cancer (TNBC) cells, and that the ARRB1 expression level was negatively correlated with tumor histological grade and positively correlated with the survival of patients with TNBC. These results suggested that ARRB1 may have a tumor-suppressive effect in breast cancer, a conclusion that was further supported in a study by Xiong et al. (Xiong et al., 2020). In contrast, a different study reported that ARRB1 mediated the metastatic growth of breast cancer cells by promoting VEGF expression (Shenoy et al., 2011), suggesting that ARRB1 may play dual and opposing roles in breast cancer. Together, these results suggest that ARRB1 plays different roles in different tumors, even in different biological processes of the same tumor.

ARRB1 expression has some prognostic value in several cancers, among which KIRC and LUAD drew our attention. ARRB1 was not only associated with a good prognosis in patients with KIRC and LUAD but was also lowly expressed in tumor tissue. In addition, correlation analysis of independent GEO cohorts also demonstrated relatively high expression of ARRB1 in low-stage LUAD and low-grade KIRC, which together suggested the possibility of ARRB1 as a tumor suppressor gene in KIRC and LUAD. Interestingly, the current studies on the prognostic value of ARRB1 in lung cancer have produced conflicting results. A report that corroborates with our results suggests that the deletion of ARRB1 expression predicted an unfavorable prognosis in non-small cell lung cancer (Ma et al., 2016). However, in another study recruiting 105 patients with surgically resected lung adenocarcinoma, overexpression of ARRB1 was considered to be associated with an unfavorable prognosis in lung adenocarcinoma (Qiu et al., 2015). These conflicting results may reflect patients with different genetic backgrounds or molecular types in lung cancer and still need further validation in future cohorts with larger samples. Although ARRB1 expression is low in most tumor tissues, several studies have suggested that ARRB1 may be a marker of poor prognosis in a small proportion of cancers. For instance, in one study, ARRB1 protein expression was found to be upregulated in gallbladder cancer (GBC) tissue and correlated with aggressive clinicopathological features and poor prognosis in patients (Zhang et al., 2021). In a different study, meanwhile, the authors noted that ARRB1 influenced the metastasis of hepatocellular carcinoma *via* extracellular signal-regulated kinase-mediated EMT (Xu et al., 2020). Similarly, ARRB1-mediated inhibition of FOXO3a was reported to contribute to the growth of prostate cancer cells both *in vitro* and *in vivo*, an effect that was likely exerted *via* the promotion of cell proliferation and EMT (Kong et al., 2018). Combined, these results suggest that the detection of ARRB1 in a subset of tumors may help predict the prognosis of affected patients and that targeted modulation of ARRB1 may represent an effective therapeutic strategy for the treatment of these tumors.

The function of immune cells in the tumor microenvironment can significantly influence tumor prognosis. To further explore the potential role of ARRB1 in the tumor immune microenvironment, we first determined whether a correlation existed between ARRB1 and immune cell infiltration. The results showed that ARRB1 expression in KICH, KIRP, SARC, THCA, DLBC, and LUSC was positively correlated with stromal cell and immune cell scores. A significant correlation was also detected between ARRB1 expression in DLBC and M1 macrophage contents and memory B cells. To date, no studies on ARRB1 in DLBC have been reported. However, the role of ARRB1 in DLBC merits further exploration given the high levels of expression observed for ARRB1 in DLBC in this study. While analyzing putative correlations between ARRB1 expression and three types of immunomodulators, we observed that ARRB1 expression in KICH was significantly and positively correlated with various immunosuppressive agents, including CD244, CSF1R, LGALS9, and PDCD1LG2. CD244 is a member of the signaling lymphocyte activating molecule (SLAM) family of proteins, and the binding of CD244 to its ligand CD48 on neighboring cells delivers signals, both stimulatory and inhibitory, that regulate immune function (Pahima et al., 2019; Sun et al., 2021). For instance, CD244 can mediate immune escape in mice with melanoma by regulating CD4^+^ and CD8^+^ T-cell expression (Goding et al., 2013). Meanwhile, the colony-stimulating factor-1 receptor (CSF-1R) signaling pathway can contribute to the immunosuppressive tumor microenvironment, and its inhibitors are currently being investigated in clinical trials (Riaz et al., 2021). In addition, exosome LGALS9 can regulate systemic anti-tumor immunity by inhibiting cytotoxic T-cell activation in cerebrospinal fluid and antigen presentation by dendritic cells (Wang et al., 2020).

Tumors can evade T-cell responses by losing MHC/human leukocyte antigen (HLA) class I molecules (Garrido and Aptsiauri, 2019). In KICH, the expression of ARRB1 was positively correlated with most MHC molecules and also showed a significant and positive correlation with some immunostimulants. Together, these results suggested that ARRB1 may play a positive immunomodulatory role in KICH. However, no study to date has investigated the role of ARRB1 in solid tumors of the kidney. Accordingly, the mechanism underlying the immune-related activity of ARRB1 in KICH requires further investigation given the results obtained in our study. Interestingly, we observed that a positive correlation exists between ARRB1 and most MHC molecules in LUSC, while the opposite is true in LUAD, suggesting that the immunomodulatory effects of ARRB1 differ according to the lung cancer type. GSEA indicated that high ARRB1 expression levels in LUSC and KICH were positively involved in the activation of the immune response, adaptive immune response, adaptive immune response based on the somatic recombination of immune receptors built from immunoglobulin superfamily domains, and adenylate cyclase modulating G protein-coupled receptor signaling pathway. The results further suggested that ARRB1 may play an important immunomodulatory role in both KICH and LUSC; however, the associated mechanism of action remains unknown, and merits further investigation.

Over the past decade, ICIs have become an important tool in cancer treatment, and effective efficacy-predicting biomarkers are key to improving responses to immunotherapy. In this study, two biomarkers of immunotherapy (TMB and MSI) showed a significant association with ARRB1 expression in some cancers. The rationale for using TMB and MSI as biomarkers for ICI is similar: many mutations produce altered peptides that are processed by the MHC, thereby generating neoantigens that prompt the immune system to mount an anti-tumor response. Thus, there is an overlap between MSI-high/mismatch repair-deficient tumors and a high TMB in some cases (Goodman et al., 2019). In this study, the correlation of ARRB1 with TMB and MSI was consistent across a variety of tumors. ARRB1 was positively correlated with TMB and MSI in ESCA but was negatively correlated with these two parameters in BRCA, THCA, STAD, READ, PRAD, and LUSC, implying that in patients with these cancers with high ARRB1 expression, immune cell targeting neoantigens are less frequent and the efficacy of immune checkpoint inhibitors may be diminished as a result. Immune checkpoints operate as immunosuppressive signals, and when overexpressed, they can convey “off” signals to decrease immune response, allowing tumor cells to elude immune destruction. In addition, immune checkpoints operate as immunosuppressive signals, and when overexpressed, they can convey “off” signals to decrease immune response, allowing tumor cells to elude immune destruction (Pardoll, 2012). Among them, PD-L1, as an important Immune checkpoint, is not only the main target of ICIs therapy, but can also indicate the efficacy of ICIs therapy in some cases (Yi et al., 2018). Our result suggests a broad association of ARRB1 with PD-L1 in tumors. Together, these results suggest that ARRB1 may be an important factor influencing or predicting the response of cancer patients to immunotherapy. To further validate the effect of ARRB1 on the response to ICI therapy, we analyzed the correlation between ARRB1 expression and the efficacy of three clinical cohorts receiving immunotherapy. Although there were no positive results in the renal cell carcinoma and advanced uroepithelial carcinoma cohorts, we found significantly low expression of ARRB1 in the responder group in the metastatic melanoma anti-PD1 therapy cohort. This suggests the potential of ARRB1 to predict the efficacy of anti-PD1 therapy in melanoma. Given that next-generation sequencing is widely used in tumor molecular typing to assist in diagnosis and treatment decision-making, clinical research on ARRB1 predicting the efficacy of immune checkpoint inhibitors deserves further exploration.

This is the first report to prospectively reveal the prognostic and therapeutic value of ARRB1 in 33 cancers. Our results suggest that ARRB1 may serve as a promising prognostic biomarker in cancers such as KIRC and LUAD. Furthermore, ARRB1 is widely associated with indicators related to the tumor immune microenvironment in a variety of cancers and may be an important factor influencing or predicting the efficacy of immunotherapy. In conclusion, this study provides valuable insights into the role of ARRB1 in cancer and provides a theoretical basis for investigating the potential mechanisms underlying the relevance of ARRB1 expression to tumor prognosis and immune regulation. Elucidating the mechanisms of ARRB1 regulation in the tumor immune microenvironment is a promising direction for the future.

## Data Availability

The datasets presented in this study can be found in online repositories. The names of the repository/repositories and accession number(s) can be found in the article/Supplementary Material.
