# Corrigendum: Role of ARRB1 in prognosis and immunotherapy: a pan-cancer analysis

**DOI:** 10.3389/fmolb.2025.1577764

**Published:** 2025-02-25

**Authors:** Yingquan Ye, Haili Jiang, Yue Wu, Gaoxiang Wang, Yi Huang, Weijie Sun, Mei Zhang

**Affiliations:** ^1^ Oncology Department of Integrated Traditional Chinese and Western Medicine, The First Affiliated Hospital of Anhui Medical University, Hefei, China; ^2^ The Traditional and Western Medicine (TCM)-Integrated Cancer Center of Anhui Medical University, Hefei, China; ^3^ Department of Infectious Diseases, The First Affiliated Hospital of Anhui Medical University, Hefei, China

**Keywords:** ARRB1, pan-cancer, prognosis, tumor immunity, biomarker

In the published article, there was an error in the **Funding** statement. The funding number was incorrect. The correct **Funding** statement appears below.

The authors apologize for this error and state that this does not change the scientific conclusions of the article in any way. The original article has been updated.

