# Supplementary figures and images for: Role of ARRB1 in prognosis and immunotherapy: A Pan-Cancer analysis
Source: Front Mol Biosci. 2022 Sep 23;9:1001225. doi: 10.3389/fmolb.2022.1001225 (PMC9538973; doi:10.3389/fmolb.2022.1001225)

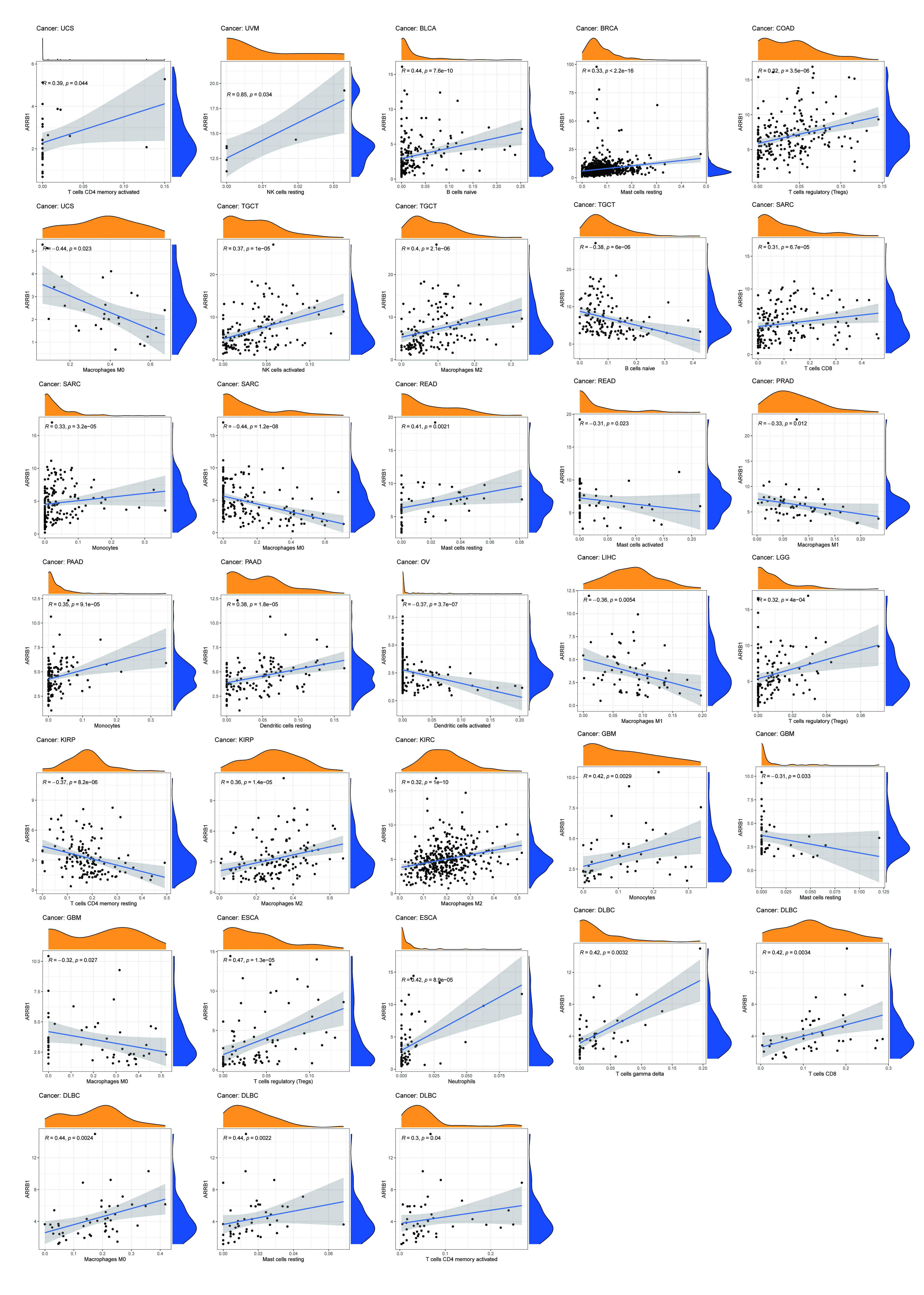

Supplement: Supplementary file 1 [file Image3.jpeg]

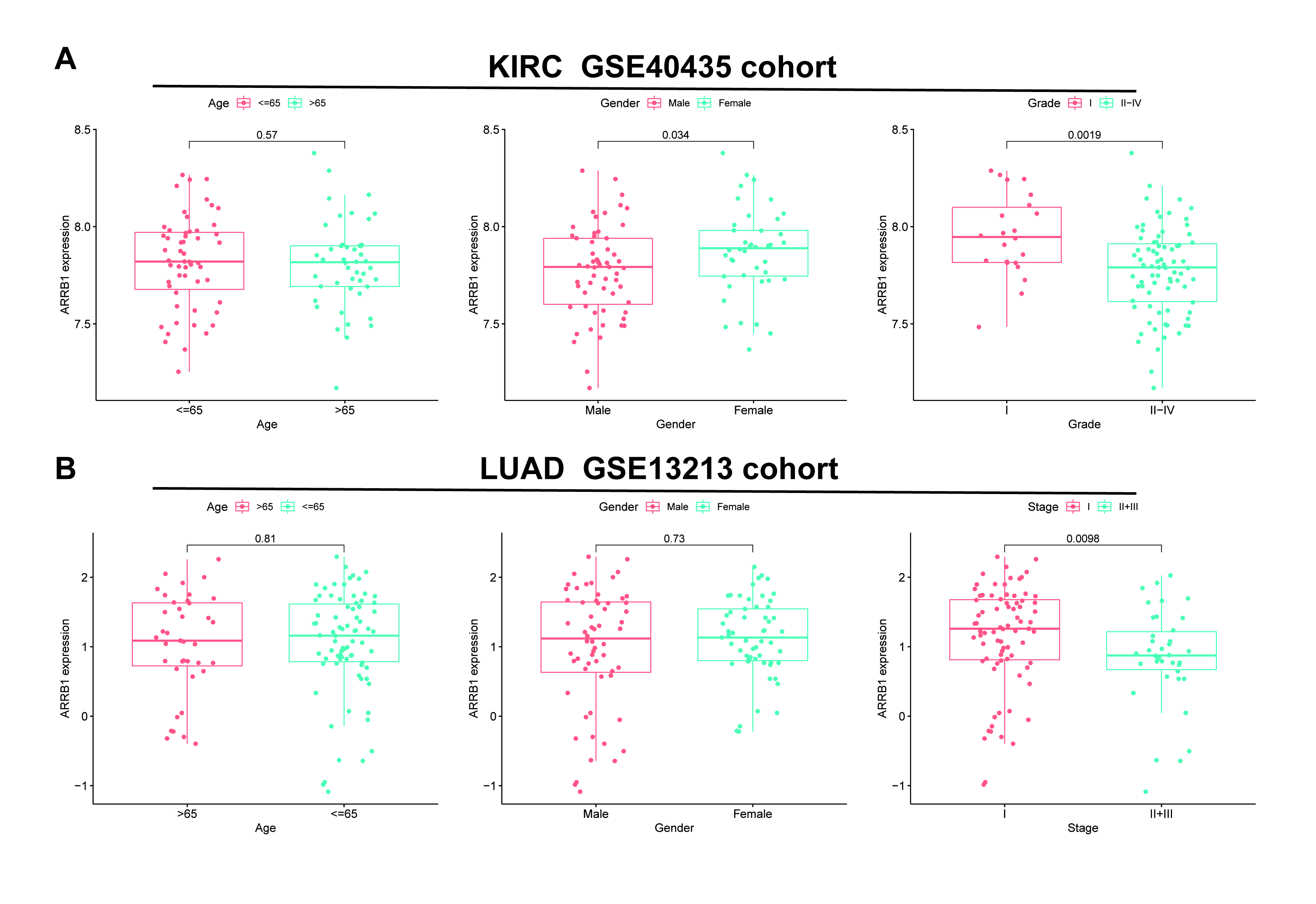

Supplement: Supplementary file 2 [file Image1.jpeg]

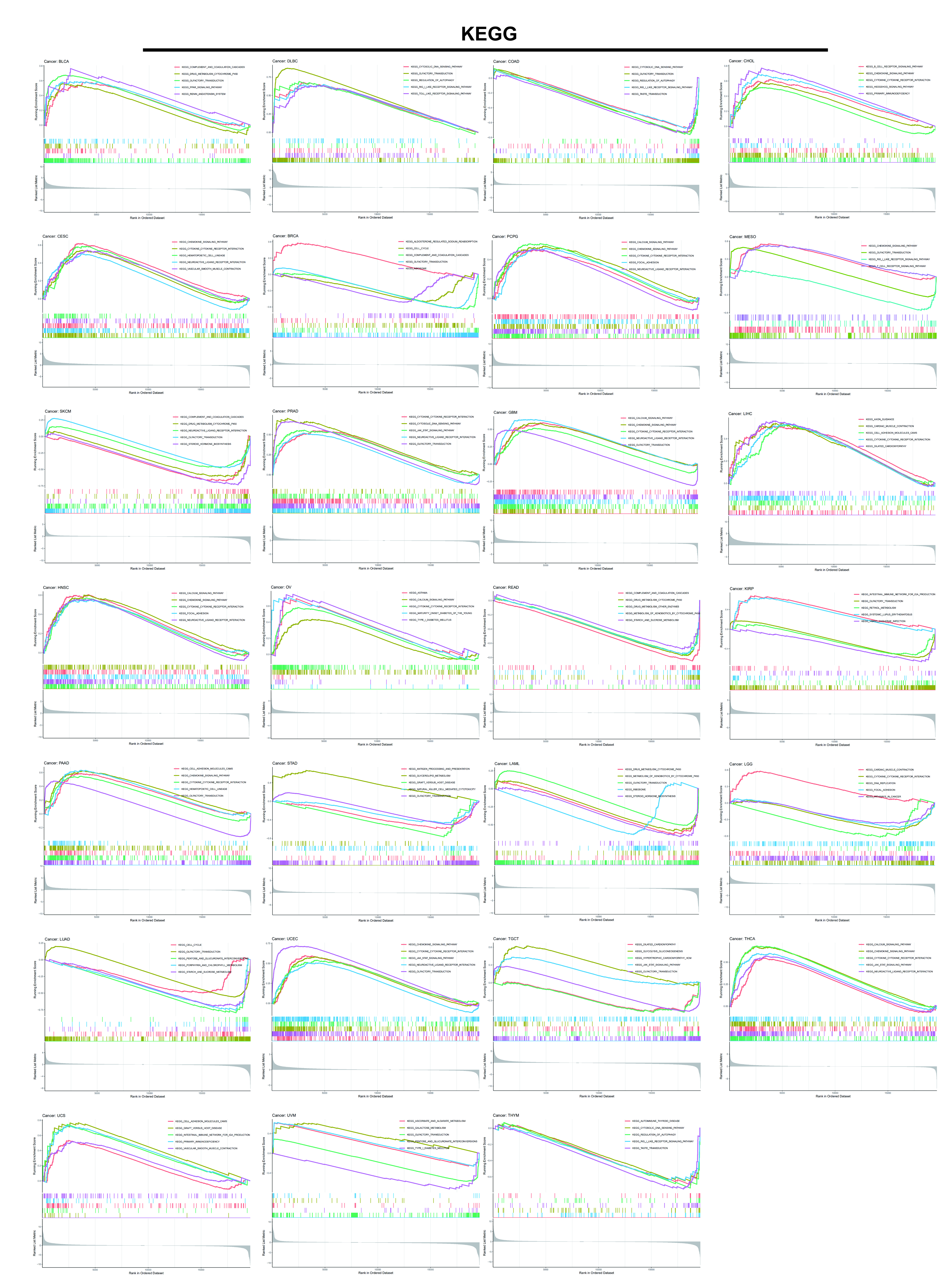

Supplement: Supplementary file 3 [file Image4.jpeg]

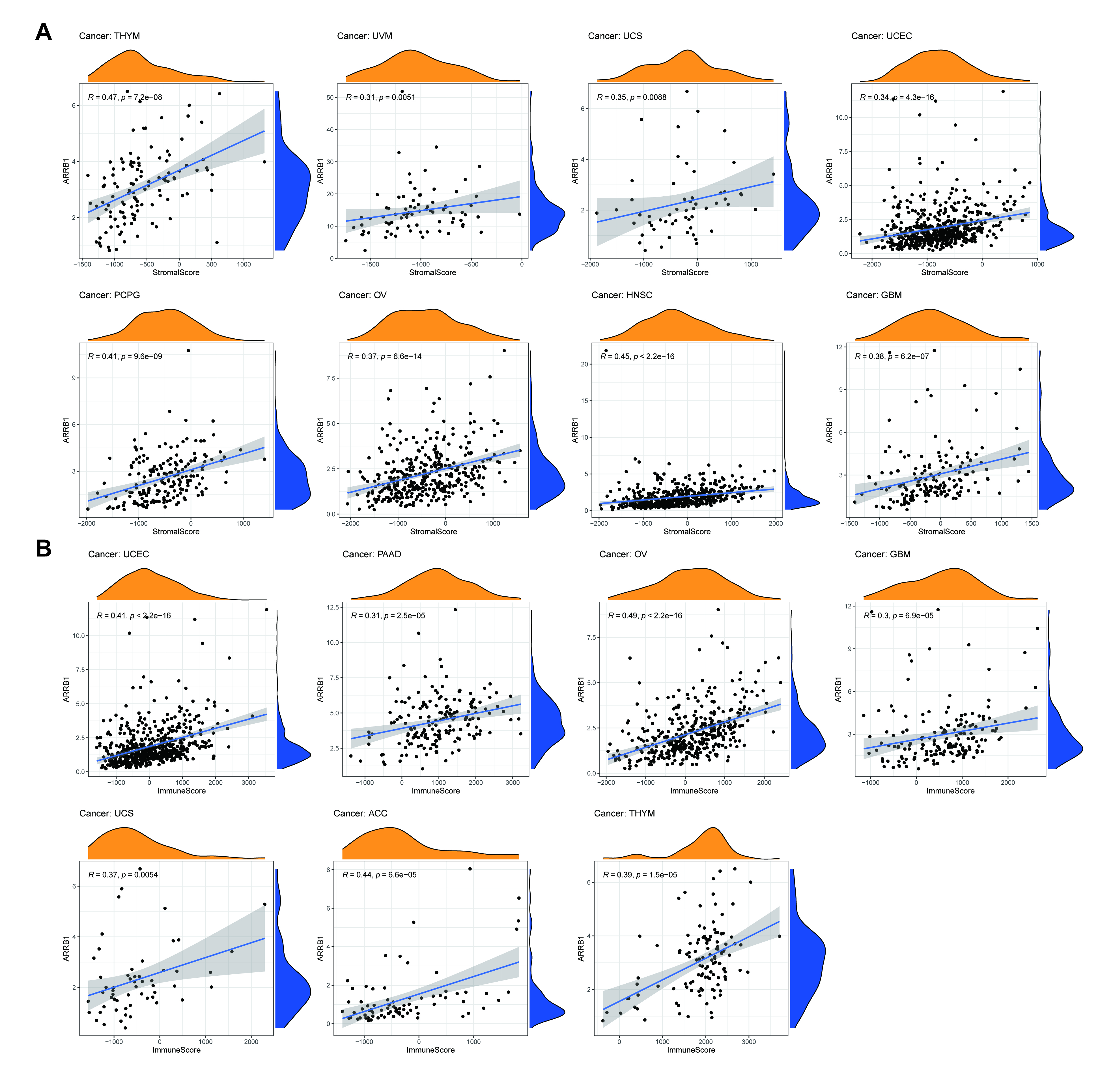

Supplement: Supplementary file 5 [file Image2.jpeg]

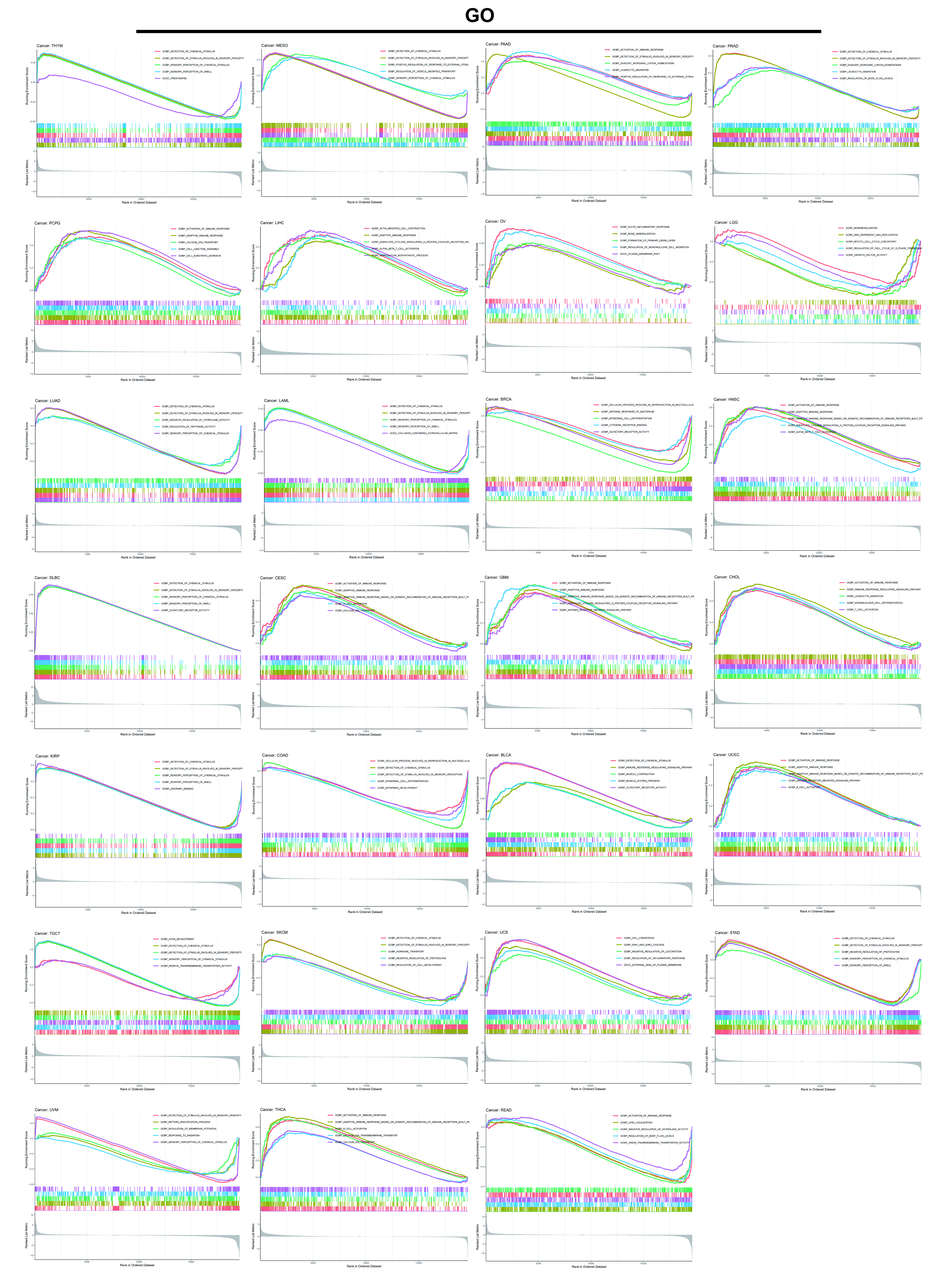

Supplement: Supplementary file 6 [file Image5.jpeg]
