# Supplementary material for: Role of ARRB1 in prognosis and immunotherapy: A Pan-Cancer analysis
Source: Front Mol Biosci. 2022 Sep 23;9:1001225. doi: 10.3389/fmolb.2022.1001225 (PMC9538973; doi:10.3389/fmolb.2022.1001225)
Supplement: Supplementary file 4 [file DataSheet1.ZIP › Supplementary Materials/Supplementary Figure legend.docx]

**FIGURE S1 |** Correlation of ARRB1 mRNA expression with clinicopathological parameters in KIRC and LUAD. **(A)** Correlation of ARRB1 expression with clinicopathological parameters (age, gender, and tumor grade) of KIRC in the GSE40435 cohort; **(B)** Correlation of ARRB1 expression with clinicopathological parameters (age, gender, and tumor stage) of LUAD in the GSE13213.

**FIGURE S2 |** Correlation between ARRB1 expression and ESTIMATE score and immune cell infiltration. **(A)** Correlation between ARRB1 expression and stromal cell score in tumor tissue; **(B)** Correlation between ARRB1 expression and immune cell score in tumor tissue; **(C)** Correlation between ARRB1 expression and immune cell infiltration. Only correlation plots with correlation coefficients *R*>0.5 and *p*<0.001 are illustrated.

**FIGURE S3 |** Correlation between ARRB1 expression and ESTIMATE score and immune cell infiltration. **(A)** Correlation between ARRB1 expression and stromal cell score in tumor tissue; **(B)** Correlation between ARRB1 expression and immune cell score in tumor tissue; **(C)** Correlation between ARRB1 expression and immune cell infiltration. Only correlation plots with correlation coefficients *R*>0.5 and *p*<0.001 are illustrated.

**FIGURE S4 |** GSEA of ARRB1 expression in cancers. **(A)** GO enrichment plots by the ARRB1 expression from GSEA analysis. **(B)** KEGG enrichment plots by the ARRB1 expression.

**FIGURE S5 |** GSEA of ARRB1 expression in cancers. **(A)** GO enrichment plots by the ARRB1 expression from GSEA analysis. **(B)** KEGG enrichment plots by the ARRB1 expression.
