# Supplementary material for: Role of ARRB1 in prognosis and immunotherapy: A Pan-Cancer analysis
Source: Front Mol Biosci. 2022 Sep 23;9:1001225. doi: 10.3389/fmolb.2022.1001225 (PMC9538973; doi:10.3389/fmolb.2022.1001225)
Supplement: Supplementary file 4 [file DataSheet1.ZIP › Supplementary Materials/List of Supplementary Materials.doc]

# Supplementary Materials

1. Appendix 1. ARRB1 expression data in 33 tumors

2. Appendix 2. Patient clinical parameters

3. Appendix 3. Single-sample gene set enrichment analysis (ssGSEA) scores

4. Appendix 4. ARRB1-associated gene set

5. Appendix 5. Merged data on ARRB1 expression and patient survival

6. Appendix 6. univariate Cox regression analysis data

7. Appendix 7. ESTIMATE Score analysis data

8. Appendix 8. Correlation data between ARRB1 expression and immune score and stromal score

9. Appendix 9. CIBERSORT algorithm data

10. Appendix10. Data on the correlation between ARRB1 expression and immune infiltrating cells

11. Appendix 11. Data on tumor mutation burden (TMB)

12. Appendix 12. Correlation data between ARRB1 expression and TMB

13. Appendix 13. Data on microsatellite instability (MSI)

14. Appendix 14. Correlation data between ARRB1 expression and MSI

15. Appendix 15. Correlation data between ARRB1 expression and PD-L1
